# The Magic Methyl and Its Tricks in Drug Discovery and Development

**DOI:** 10.3390/ph16081157

**Published:** 2023-08-15

**Authors:** Pedro de Sena Murteira Pinheiro, Lucas Silva Franco, Carlos Alberto Manssour Fraga

**Affiliations:** 1 Laboratório de Avaliação e Síntese de Substâncias Bioativas (LASSBio), Instituto de Ciências Biomédicas, Universidade Federal do Rio de Janeiro, Rio de Janeiro 21941-902, RJ, Brazil; pedro_senamp@hotmail.com (P.d.S.M.P.); silvafrancolucas@gmail.com (L.S.F.); 2 Instituto Nacional de Ciência e Tecnologia de Fármacos e Medicamentos (INCT-INOFAR), CCS, Universidade Federal do Rio de Janeiro, Cidade Universitária, Rio de Janeiro 21941-902, RJ, Brazil; 3 Programa de Pós-Graduação em Farmacologia e Química Medicinal, Instituto de Ciências Biomédicas, Universidade Federal do Rio de Janeiro, Cidade Universitária, Rio de Janeiro 21941-902, RJ, Brazil

**Keywords:** methyl, methylation, methyl effect, magic methyl, methylation effect, drug design

## Abstract

One of the key scientific aspects of small-molecule drug discovery and development is the analysis of the relationship between its chemical structure and biological activity. Understanding the effects that lead to significant changes in biological activity is of paramount importance for the rational design and optimization of bioactive molecules. The “methylation effect”, or the “magic methyl” effect, is a factor that stands out due to the number of examples that demonstrate profound changes in either pharmacodynamic or pharmacokinetic properties. In many cases, this has been carried out rationally, but in others it has been the product of serendipitous observations. This paper summarizes recent examples that provide an overview of the current state of the art and contribute to a better understanding of the methylation effect in bioactive small-molecule drug candidates.

## 1. Introduction

The small, monovalent, and lipophilic methyl group (-CH_3_) is versatile and of great importance in the design or optimization of bioactive compounds, whether in terms of pharmacodynamic or pharmacokinetic properties [1]. Its role in drug design and hit-to-lead optimization processes is broad, including the displacement of water molecules during molecular recognition, i.e., the realization of hydrophobic interactions [2,3]; the participation in van der Waals interactions; the modulation of physicochemical properties, such as LogP and aqueous solubility [1]; and the control of the conformational properties of a given scaffold [1]. The control of the number of conformations in a given system by methylation correlates with the strategy of conformational restriction [4,5]. Other drug design strategies, such as bioisosterism [6,7,8] and homologation [9], can also benefit from methyl group insertion. During the drug discovery process, controlling conformational behavior can not only favor the adoption of a bioactive conformation, generating a potency gain for pharmacological target modulation, but can also help break to planarity and symmetry, resulting in increased aqueous solubility while increasing lipophilicity [10,11].

Other uses of the methyl group include modulating metabolic reactions by preventing their occurrence through stereoelectronic effects, by serving as a metabolic point to prevent the formation of toxic metabolites, or by modulating the metabolic profile, making molecules softer for metabolic reactions [1].

This plethora of effects mediated by the methyl group is commonly referred to as the “methyl effect”, the “methylation effect”, or even the “magic methyl” effect. It is important to mention that there are previous works that have already reviewed this topic and are published elsewhere [1,12,13,14]. The aim of this work is to provide a recent analysis of the last 10 years, with selected key examples, to highlight how the rational use of the methyl effect has evolved since the last review published by our group [1].

## 2. The Discovery of the Anticancer Drug Tazemetostat

Undoubtedly, one of the most important recent examples of the methylation effect in the design of new drugs is related to the discovery of tazemetostat (**8**). Tazemetostat (**8**) is a recently FDA-approved agent (2020) for the treatment of patients with epithelioid sarcoma [15]. Tazemetostat (**8**) acts by inhibiting enhancer of zeste homolog 2 (EZH2), preventing methylation of histone 3 at lysine 27 (H3K27), and abnormal methylation of this site is found in many cancers [16,17,18].

**Figure 1:**
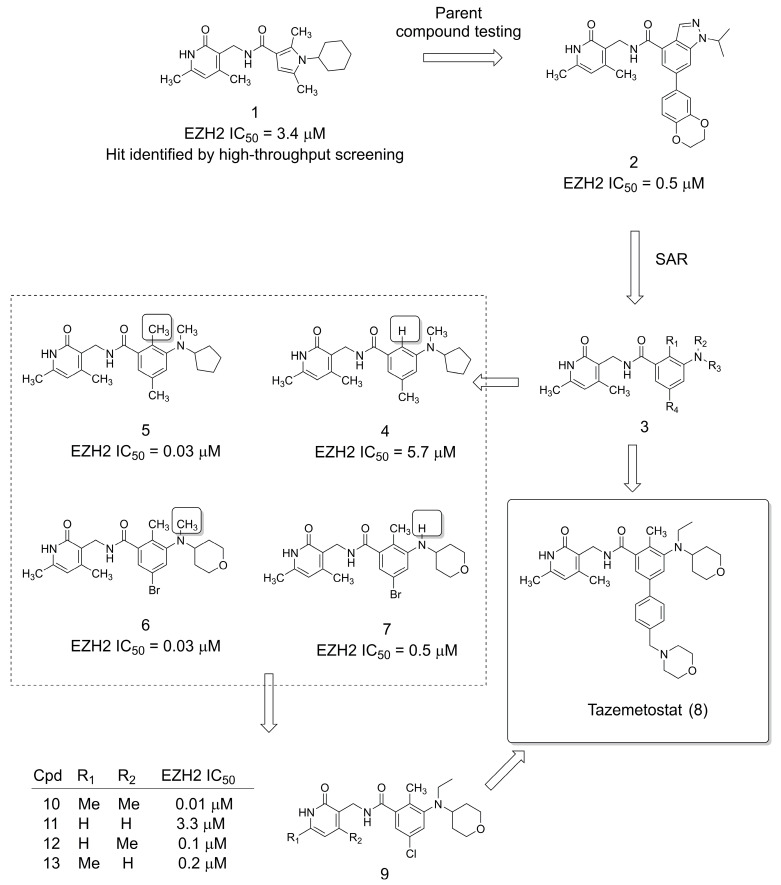
The discovery of anticancer FDA-approved drug tazemetostat (**8**) [16].

The discovery of tazemetostat (**8**) started with a high-throughput screening (HTS) campaign that resulted in the identification of the small molecule hit **1**, bearing the 4,6-dimethylpyridone moiety (Figure 1) [16]. Next, the structurally related parent compounds of **1** were screened for EZH2 inhibition, leading to the identification of **2**. Structure–activity relationship studies were performed, allowing important analyses of the methylation effect on **3**. For example, the conformational effect produced by methylation at the R1 and R2 positions of **3** was remarkable, as can be seen by comparing the methylated derivatives (**5** and **6**) and their unmethylated analogs (**4** and **7**), which presented more than a 10-fold decrease in potency. Moreover, the methylation effect at the 4,6-dimethylpyridone moiety was investigated (**9**), showing significant differences in potency (**10**–**13**), and the dimethylated compound **10** was the most potent. Subsequently, these important studies regarding the methylation pattern of this system resulted in the discovery of the drug tazemetostat (**8**) [16].

## 3. The Methylation Effect in Pharmacodynamic Optimization

### 3.1. Selective EZH2 Inhibitors

**Figure 2:**
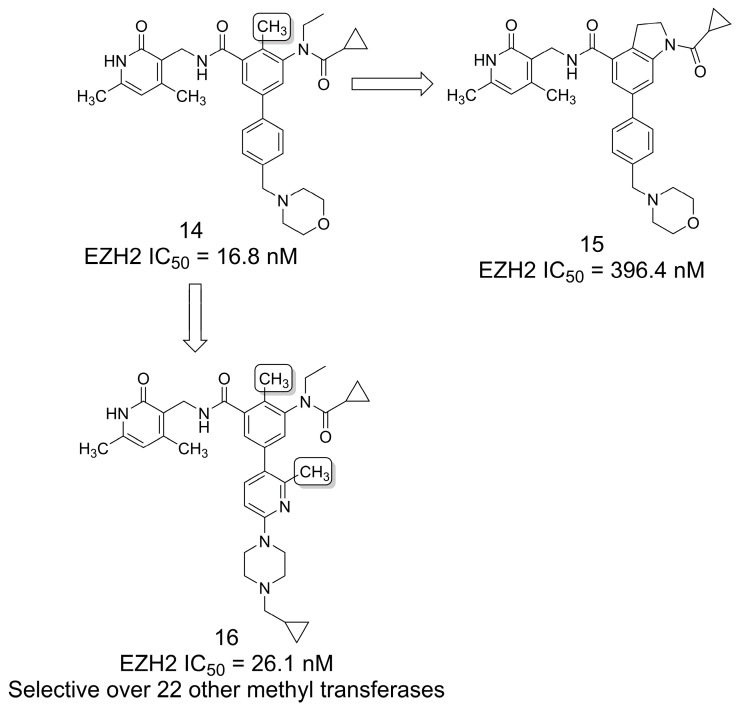
The methylation effect in the discovery of EZH2-selective inhibitors [19].

Aiming to obtain new selective EZH2 inhibitors related to tazemetostat (**8**), the authors of [19] first tried to perform the cyclization of compound **14** by linking the methyl and ethyl groups to form an indoline ring (**15**) (Figure 2). However, it was observed that this modification led to a loss in potency against the EZH2 wild-type enzyme and the EZH2 Y641F mutant. The authors suggest that the cyclization abolished the “magic methyl” effect previously reported to be key to the FDA-approved drug **8**. Therefore, the authors selected the open-ring analog (**14**) for further structure–activity relationship (SAR) exploration, resulting in compound **16**, a derivative with a second methyl group at the pyridine ring that showed selectivity over 22 other methyl transferases [19].

### 3.2. PI3K/mTOR Inhibitors

**Figure 3:**
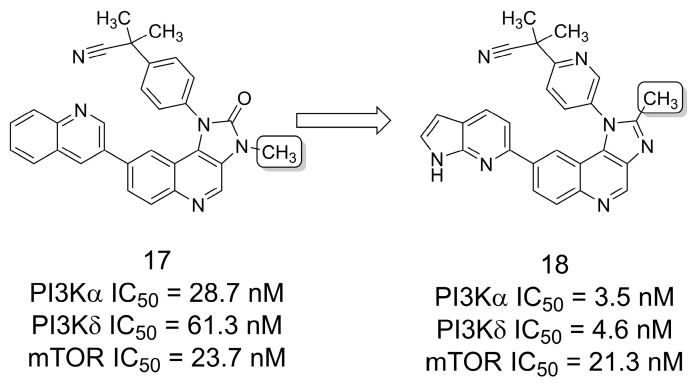
The methylation effect in the design of PI3K/mTOR inhibitors [20].

A series of 2-methyl-1*H*-imidazo[4,5-*c*]quinolines were reported [20] based on ring bioisosterism with the 1,3-dihydro-2*H*-imidazo[4,5-*c*]quinolin-2-one system present in the phosphoinositide 3-kinases (PI3Ks) and mammalian target of rapamycin (mTOR) inhibitor and clinical candidate BEZ235 (**17**) [21] (Figure 3). Targeting the PI3K/AKT/mTOR pathway is a validated strategy for cancer treatment because it is aberrantly activated in several human cancers and plays an essential role in cell growth, proliferation, differentiation, and apoptosis [22,23]. The rationale for the modification was to utilize the methyl group to preserve cell permeability and cell absorption capacity while reducing the number of polar heteroatoms (i.e., the oxygen atom of the carbonyl group of candidate **17**). Hence, a series of compounds were synthesized in order to explore the potential of the 2-methyl-1*H*-imidazo[4,5-*c*]quinoline scaffold and to improve its drug-like profile. This study resulted in compound **18** having the best profile of kinase selectivity, cellular antiproliferative activity, western blot and immunohistochemical analyses, antitumor efficacy *in vivo*, and pharmacokinetic properties [20].

### 3.3. Selective κ-Opioid Receptor Antagonists

**Figure 4:**
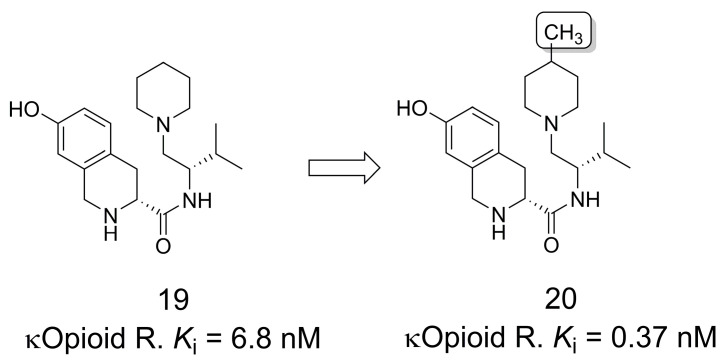
The methylation effect in the discovery of selective κ opioid receptor antagonists [26].

Tetrahydroisoquinoline derivatives have been described as selective κ-opioid receptor antagonists and as compounds of interest for the treatment of several CNS disorders, such as substance abuse, depression, and anxiety [24]. Compound **19** was first discovered as a potent antagonist of this receptor [25], and subsequent SAR evaluations were performed that focused, among other modifications, on the study of the methylation pattern of the piperidine ring. The results revealed that the 4-methylated analog (**20**) had an 18-fold increase in the affinity for κ-opioid receptors compared to **19** [26] (Figure 4).

### 3.4. Cannabinoid Receptor Modulators

**Figure 5:**
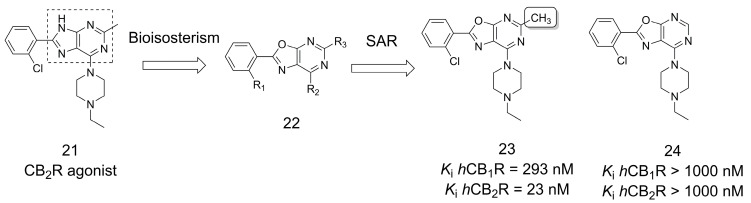
Design of oxazolo[5,4-*d*]pyrimidines series as new CB1/CB2 receptor modulators [29].

Modulation of the endocannabinoid system by targeting G-protein-coupled cannabinoid receptors has broad therapeutic applications ranging from pain to cancer treatment [27,28]. A series of oxazolo[5,4-*d*]pyrimidines (**22**) were designed via the bioisosterism strategy as new cannabinoid receptor 1 (CB_1_R) and cannabinoid receptor 2 (CB_2_R) modulators (Figure 5) [29]. A classical bioisosteric heterocycle replacement strategy was applied to compound **21**, a CB_2_R agonist developed by Eli Lilly [30]. SAR studies revealed the importance of methylation at position 5 of this core when **23** was compared with the unmethylated derivative of the series (**24**). Compound **23** was characterized as a selective CB_2_R antagonist with high binding affinity in the low nanomolar range [29].

**Figure 6:**
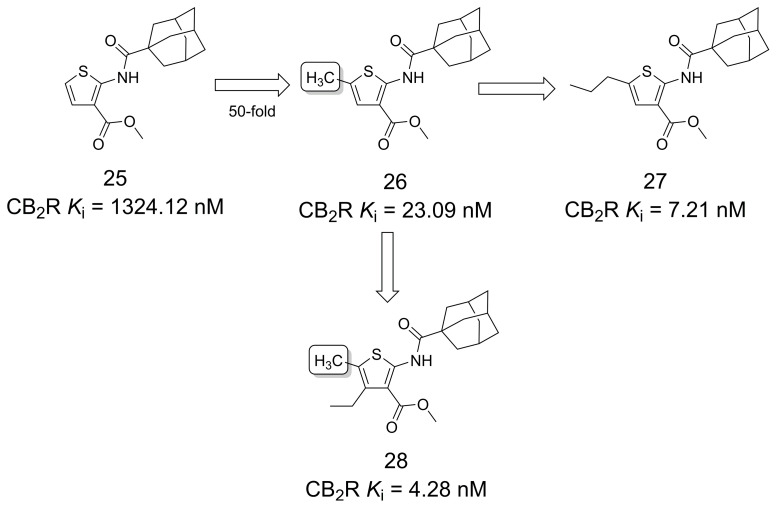
Design of selective CB_2_R agonists as potential agents for the treatment of skin inflammatory disease [31].

Mugnaini et al. [31] reported that 2-(1-adamantanylcarboxamido)thiophene derivatives (**25**–**28**) are selective CB_2_R agonists (Figure 6). The chemical starting point, compound **25**, had weak activity against CB_2_R, and the simple addition of the methyl group (**26**) resulted in a 50-fold increase in the affinity. The authors remarked on the crucial role that methyl groups play in biologically active small molecules and emphasized that the effect was likely due to **26**’s ability to successfully insert its methyl into the receptor binding site to establish effective hydrophobic contacts. This theory was supported by the fact that the n-propyl analog (**27**) obtained only a threefold increase in the affinity for CB_2_ receptors. These results are in stark contrast to studies suggesting that adding a methyl group to a lead molecule can result in a 10-fold increase in activity in only 8% of cases, while a 100-fold increase in potency is much less likely, occurring in 0.4% of cases [14,31].

**Figure 7:**
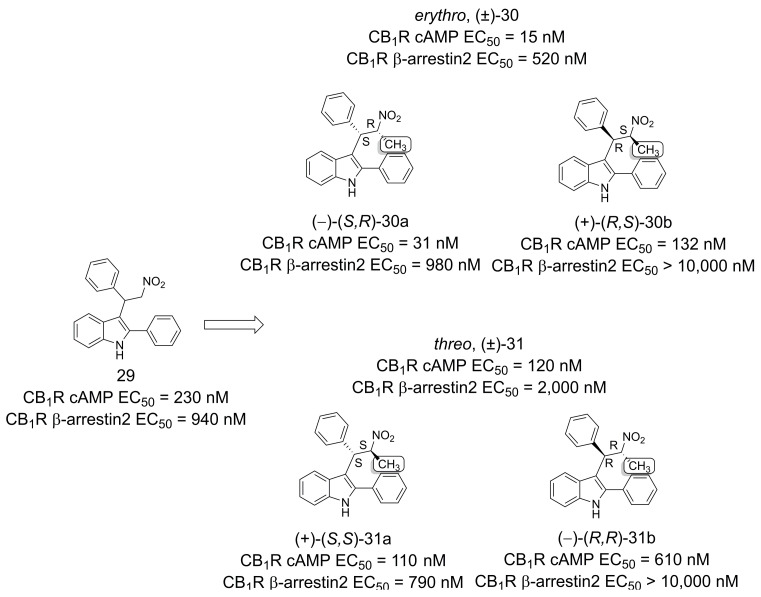
Methylation effect on the design of modulators for CB_1_R [32].

Garai and colleagues [32] employed the magic methyl effect to increase the potency and efficacy of GAT211 (**29**) [33], a cannabinoid type-1 receptor (CB_1_R) agonist-positive allosteric modulator (ago-PAM) (Figure 7). The strategic placement of a methyl group at the *alpha* position of the nitro functional group was hypothesized to be advantageous in terms of activity and functional selectivity, as it generated two diastereomers and an additional chiral center. Results from studies with the two diastereomers highlighted the increased potency and efficacy of *erythro*, (±)-**30** compared to *threo*, (±)-**31**. The analysis of the separate enantiomers highlighted (−)-(*S*,*R*)-**30a** and (+)-(*S*,*S*)-**31a** as the most potent. This result represents the first example of a diastereoselective CB_1_R allosteric modulator interaction [32].

### 3.5. Histamine 1 Receptor Antagonists

**Figure 8:**
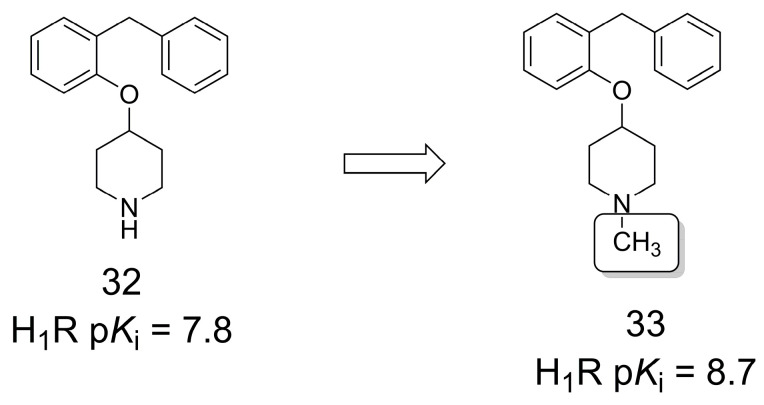
Methylation effect on fragment-like discovery of H_1_R antagonists [37].

To identify new fragment-like [34,35] histamine 1 receptor (H_1_R) antagonists, a virtual screening campaign was performed, which led to the identification of compound **32** (Figure 8) [36]. Next, **32** was used for SAR exploration and to investigate the role of the well-defined receptor binding pockets, i.e., (1) the amine binding region, (2) the upper and lower aromatic binding regions, and (3) the effect of binding site (de)solvation on H_1_R antagonist binding. Molecular modeling analysis combined with SAR exploration indicated the amine binding region as the primary binding hotspot, preferentially binding small tertiary amines, which is related to hydrophobic interactions. The methylation effect is clear when comparing **32** and **33**, since the *N*-methylation strongly increased the binding affinity for H_1_ receptors [37].

### 3.6. Inhibitors of Phosphopantetheine Adenylyltransferase

**Figure 9:**
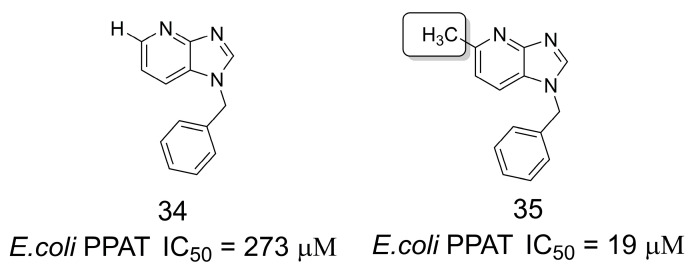
Fragment-based drug discovery of phosphopantetheine adenylyltransferase inhibitors [38].

In a study conducted by Novartis [38], a fragment-based screening approach [34,35] was used to identify inhibitors of phosphopantetheine adenylyltransferase (PPAT) for the discovery of new antibiotics for the treatment of infections caused by multidrug-resistant and pan-drug-resistant Gram-negative bacteria [38]. Fragment **34** was one of the identified hits, and hit-to-lead optimization based on C5 methylation of the imidazo[4,5-*b*]pyridine core resulted in **35**, which had a 15-fold increase in potency (Figure 9), related to additional interactions with a hydrophobic pocket (V135, M105, and L131) of the target [38].

**Figure 10:**
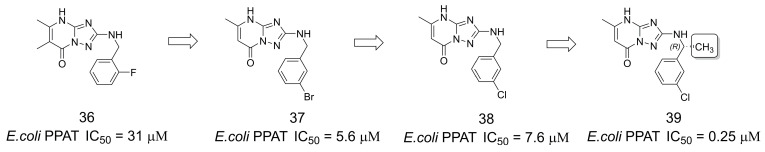
The methylation effect in fragment optimization [38].

Moreover, another fragment hit (**36**) was optimized, and the methylation pattern of this hit profoundly altered its potency (Figure 10). An X-ray cocrystal of the structurally related hit **37** revealed that this triazolopyrimidinone is bound in a similar manner to **34**. The bioisosteric replacement of the bromine atom of **37** by a chlorine atom (**38**) did not change potency significantly. Surprisingly, substitution of the benzylic position of the benzylamine with a methyl group resulted in a 30-fold activity boost, as observed for the (*R*)-methyl analog **39**, which is probably related to hydrophobic interactions [38].

### 3.7. Genetic Depletion of the Mitotic Aurora Kinase B (AURKB)

**Figure 11:**
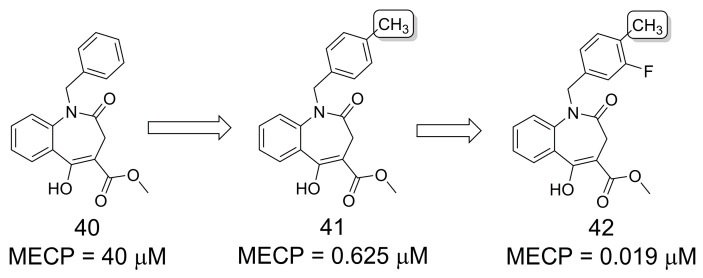
The methylation effect in polyploidy-inducing activity correlated to genetic depletion of AURKB [40].

AURKB is a gene encoding mitotic Aurora Kinase B that is overexpressed in some tumor cells, making it an interesting therapeutic target [39]. Huang and colleagues [40] employed a methyl group scanning strategy to enable hit-to-lead optimization (Figure 11) of compounds identified by mechanism-informed phenotypic screening [41], evaluating the genetic depletion of Aurora Kinase B (AURKB) [40]. The authors modified the benzene ring of hit **40** and synthesized *ortho*-, *meta*-, and *para*-methyl-substituted analogs. The *para*-substituted compound (**41**) demonstrated the best polyploidy-inducing activity, with a minimum effective concentration for polyploidy (MECP) of 0.625 μM. The authors further optimized lead **41**, resulting in compound **42** (MECP = 0.019 μM). This compound displayed substantial cytotoxic activity in several cancer cell lines and promoted the loss of function in Aurora Kinase B (AURKB) phenotypes [40].

### 3.8. Neurokinin-3 Receptor Antagonists

**Figure 12:**
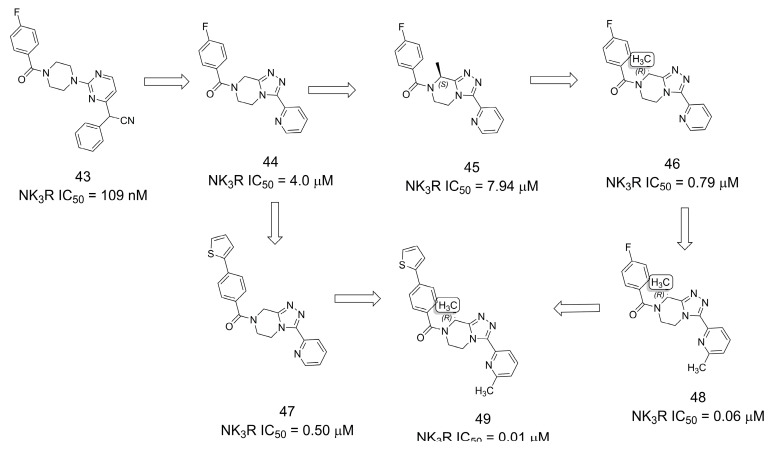
Methylation effect in the discovery of antagonists of the neurokinin-3 receptor (NK3R) [42].

The discovery of new neurokinin-3 receptor (NK_3_R) antagonists for the treatment of sex hormone disorders was described [42]. Starting with an HTS campaign, hit **43** was identified as an interesting starting point for optimization, but several issues such as poor solubility, microsomal stability, and off-target safety profile led to the selection of the parent compound **44** as the starting point (Figure 12). Despite being significantly less active, **44** presented improved off-target and PK profiles, making it more suitable for optimization. From the SAR analysis, it was possible to perceive the methylation effect by installing a methyl group at the 8-position of the tetrahydro-[1,2,4]triazolo[4,3-*a*]pyrazine core, where (*R*)-**46** presented increased potency, reaching the nanomolar scale. A second methylation at C6 of the terminal pyridine ring further increased potency, resulting in low nanomolar activity (**48** and **49**) [42].

### 3.9. Cereblon Ligands for Targeted Protein Degradation

**Figure 13:**
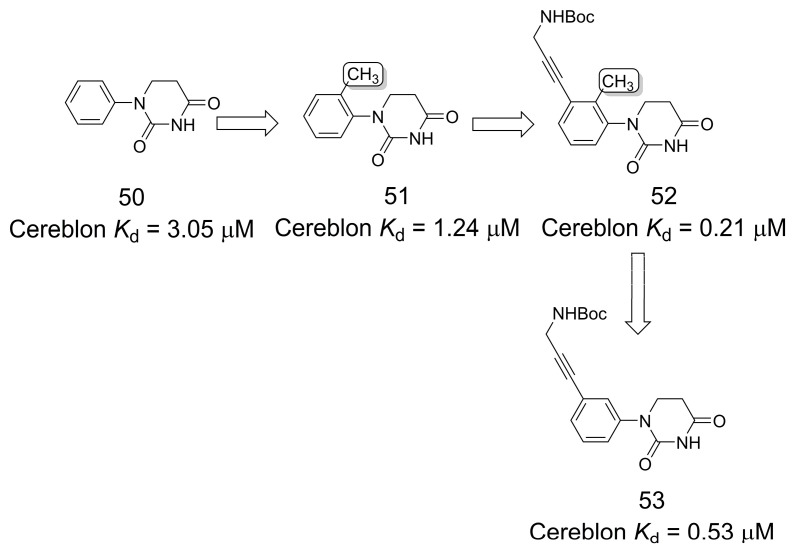
The methylation effect in new cereblon ligands for targeted protein degradation [45].

With the goal of obtaining new cereblon ligands for targeted protein degradation [43,44], Xie and coworkers [45] explored the *ortho*-effect produced by a methyl group (Figure 13). The authors modified phenyl dihydrouracil (PDHU) (**50**) (cereblon *K*_d_ = 3.05 μM) and observed that the *ortho*-substituted methyl analog (**51**) had improved binding potency (cereblon *K*_d_ = 1.24 μM). Given this result, the authors selected this compound for further modification and explored the vector at the *meta*-position for attachment of the linker and of the “protein of interest” ligand subunit. The authors identified compound **52** with the best affinity (*K*_d_ = 0.21 μM) for cereblon. The *ortho*-methylated cereblon ligands were explored and allowed the identification of potent bromodomain-containing protein 4 (BRD4) degraders [45].

### 3.10. Putative Dual Inhibitor of Tubulin and EGFR by Phenotypic Approach

**Figure 14:**
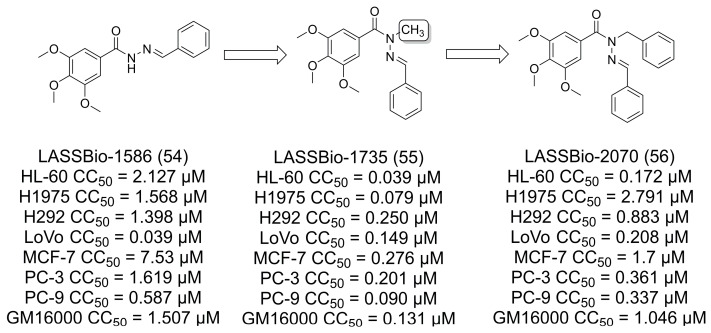
The methylation effect in combretastatin A-4 analogs [46].

Barbosa and colleagues [46] described a series of combretastatin A-4 analogs based on the *N*-acylhydrazone (NAH) LASSBio-1586 (**54**) with cytotoxic and antimitotic activity (Figure 14). Homologation [9] studies on the amide nitrogen led to the benzyl homolog of LASSBio-1586 (**54**), LASSBio-2070 (**56**), which showed microtubule-stabilizing behavior, while the methylated homolog, LASSBio-1735 (**55**), had microtubule-destabilizing behavior. In addition, none of the compounds had better cytotoxic activity when compared to the *N*-methylated compound LASSBio-1735 (**55**) [46].

### 3.11. Phosphodiesterase Inhibitors

**Figure 15:**
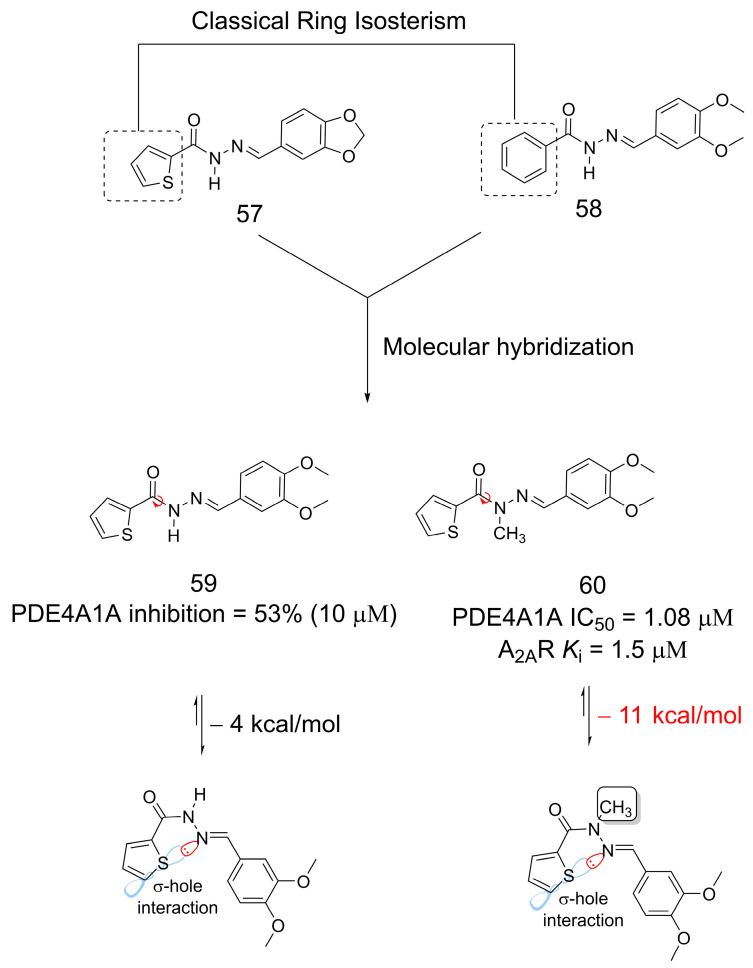
The methylation effect stabilizing bioactive conformation of multitarget *N*-acylhydrazone derivatives [50].

The methylation effect was also shown to have an impact on multitarget small molecule discovery. The compounds (**59** and **60**) were designed through molecular hybridization [47] and bioisosteric replacement [7] strategies using **57** and **58** as starting points (Figure 15). As previously reported, **57** is an adenosine A_2A_ receptor agonist [48], and **58** is a phosphodiesterase 4 (PDE4) inhibitor [49]. Insertion of the methyl group at the amide nitrogen of the *N*-acylhydrazone (**60**) moiety significantly increased the percent inhibition of PDE4A1A compared to the non-methylated analog (**59**). Further evaluation showed that **60** had an IC_50_ of 1.08 µM for PDE4A1A inhibition and a moderate affinity for the adenosine A_2A_ receptor (*K*_i_ = 1.5 µM), making this compound interesting for the treatment of pulmonary arterial hypertension. Regardless of the presence of methyl, there is a σ-hole intramolecular interaction between the sulfur atom of the thiophene ring and the nitrogen atom of the imine, which establishes the bioactive conformation for the system, as already described for *N*-acylhydrazone derivatives [50,51]. However, with the *N*-methylation of the amide, there is a greater stabilization of this conformation and, consequently, an improvement in biological activity (Figure 15) [50].

**Figure 16:**
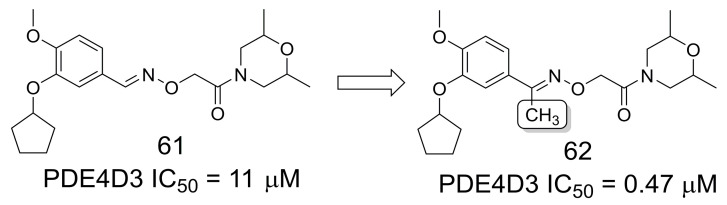
The methylation effect in the design of PDE4D inhibitors [52].

Brullo and colleagues [52] designed and synthesized methylated PDE4 inhibitors as possible candidates for Alzheimer’s disease treatment due to their role in pro-cognitive effects. The authors observed that the methylated open-chain linkers were superior to both de-methylated and cyclic conformationally constrained analogs. For example, the methylated compound **62** showed an IC_50_ of 0.47 µM (PDE4D3), and the de-methylated **61** compound had an IC_50_ of 11 μM (PDE4D3) (Figure 16). In addition, crystallographic studies showed that the methyl group was able to interact with the binding site and improve potency while maintaining the linker flexibility necessary for inhibitors to interact with PDE4 [52].

**Figure 17:**
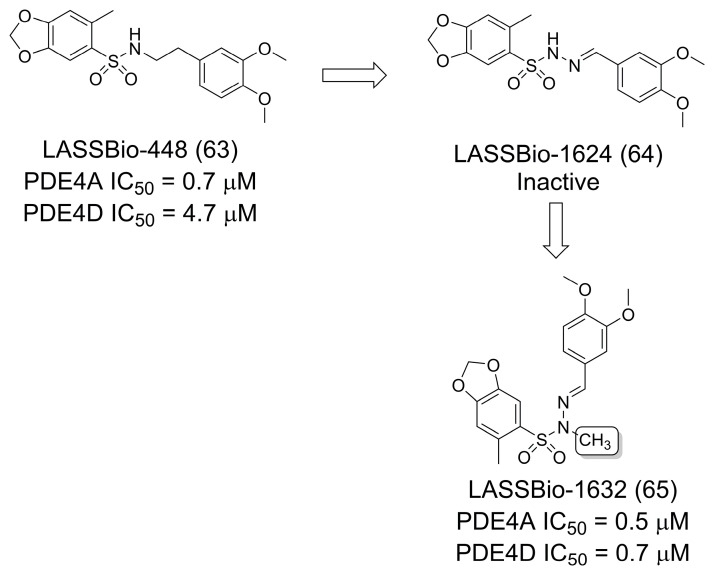
Exploration of the methylation effect for the discovery of selective PDE4A and PDE4D inhibitors [53].

Nunes et al. [53] reported the optimization of the sulfonamide prototype LASSBio-448 (**63**) [54], a PDE4 inhibitor (PDE4A IC_50_ = 0.7 μM; PDE4D IC_50_ = 4.7 μM), for the treatment of pulmonary inflammatory diseases such as asthma (Figure 17. In this work, the authors investigated the methyl effect by designing and synthesizing methylated homologs on the Nsp3 of a series of sulfonamides and sulfonylhydrazones. While the non-methylated sulfonylhydrazone LASSBio-1624 (**64**) was inactive against PDE4, the *N*-methylated sulfonylhydrazone derivative, LASSBio-1632 (**65**), was active, showing anti-asthmatic activity associated with the inhibition of PDE4A (IC_50_ = 0.5 μM) and PDE4D (IC_50_ = 0.7 μM). The authors also reported that the lead compound was able to block airway hyperreactivity and TNF-α production in lung tissue [53].

### 3.12. Rho-Associated Kinase (ROCK) Inhibitors

**Figure 18:**
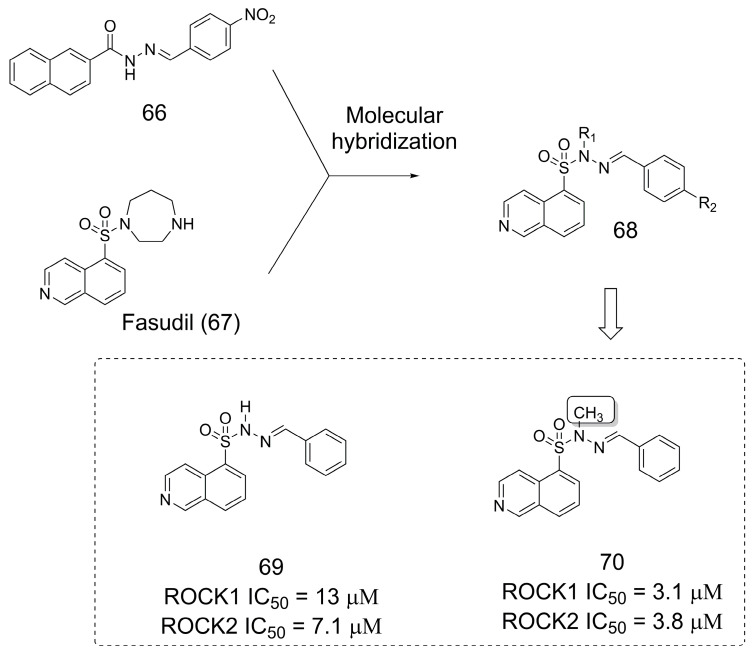
The methylation in *N*-sulfonylhydrazone derivatives [57].

ROCK inhibitors have emerged as interesting candidates for treating neurodegenerative diseases [55,56]. A series of *N*-sulfonylhydrazone derivatives were designed as Rho-associated kinase (ROCK) inhibitors [57] through molecular hybridization between the approved drug fasudil (**67**), a ROCK inhibitor [58], and **66**, a previously reported inhibitor of nuclear factor kappa-B kinase subunit beta (IKKβ) [59] (Figure 18). Within this molecular framework (**68**), **69** was discovered to have low micromolar activity for ROCK1/2 inhibition. *N*-methylation of **69** resulted in **70**, which was three- to fourfold more potent for ROCK1/2 inhibition [57].

### 3.13. Ligands of Toll-like Receptor 4/Myeloid Differentiation Protein 2 Complex

**Figure 19:**
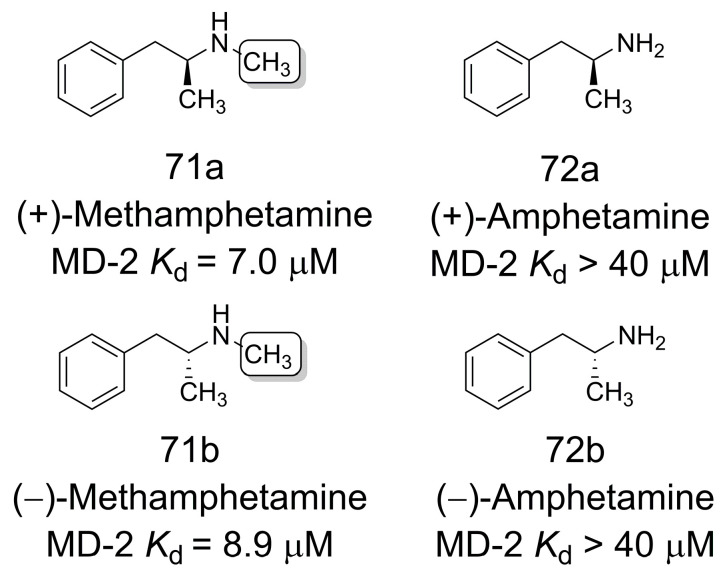
Ligands of Toll-like Receptor 4/Myeloid Differentiation Protein 2 complex [60].

Zhang and coworkers [60] demonstrated that Toll-like Receptor 4/Myeloid Differentiation Protein 2 (TLR4/MD-2) complex [61,62] recognizes methamphetamine (**71a** and **71b**) non-enantioselectively, whereas amphetamine (**72a** and **72b**) is inactive. Compared to amphetamine (**72**) (MD-2 *K*_d_ not detectable up to 40 μM), the increased TLR4/MD-2 binding affinity of methamphetamine ((+)-**71a** MD-2 *K*_d_ = 7.0 μM; (−)-**71b** MD-2 *K*_d_ = 8.9 μM) suggests that the methyl group is essential for molecular recognition (Figure 19). Molecular dynamics simulations (20 ns) and binding free energies determined by the MM-PBSA technique indicated that (+)-**71a** and (−)-**71b** had comparable binding free energies. Further energy analysis revealed that hydrophobic interactions are predominantly responsible for the binding of methamphetamine/amphetamine to TLR4/MD-2 [60].

### 3.14. Human Ghrelin Receptor Agonist

Other interesting examples of the use of methyl groups in optimization processes are macrocycles and peptides, which normally lack adequate physicochemical and pharmacokinetic properties [63,64]. In these cases, the methyl effect can be exploited to optimize these properties through conformational restriction.

**Figure 20:**
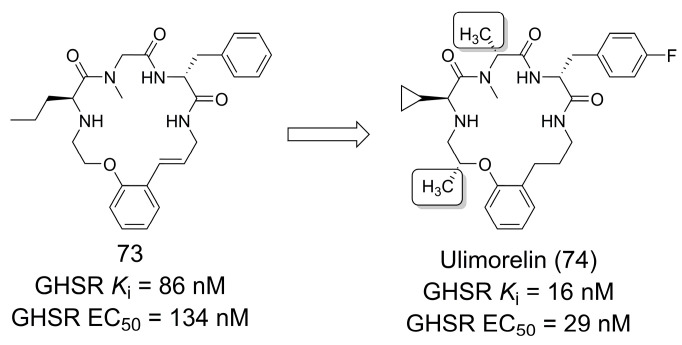
The methylation effect in the discovery of ulimorelin (**73**) [65].

A key example is the discovery of ulimorelin (**74**), a compound that has reached Phase 3 clinical trials. Ulimorelin (**74**) acts as an agonist of the human ghrelin receptor (also known as the growth hormone secretagogue receptor—GHSR) and has gastroprokinetic properties. The development of **74** was initiated by an HTS campaign that led to the identification of **73** (Figure 20). Despite its high potency, **73** did not show adequate pharmacokinetic properties, and modification of the macrocycle methylation pattern helped to stabilize the bioactive conformation of this compound, resulting in the discovery of ulimorelin (**74**), which was four- to fivefold more potent for receptor activation and showed minimally adequate pharmacokinetic properties to enter the clinical phase [65]. It is important to note that during the SAR investigation, the side chain modification of isoleucine to cyclopropyl and the introduction of *para*-fluor on the phenyl ring of **74** did not significantly affect the affinity of the compound. The authors reported that cyclopropyl is more metabolically stable than the side chain of isoleucine and that para-fluor resulted in an optimization of the ligand lipophilicity efficiency (LLE) [65].

### 3.15. Pan-Genotype NS3/4A Protease Inhibitors

**Figure 21:**
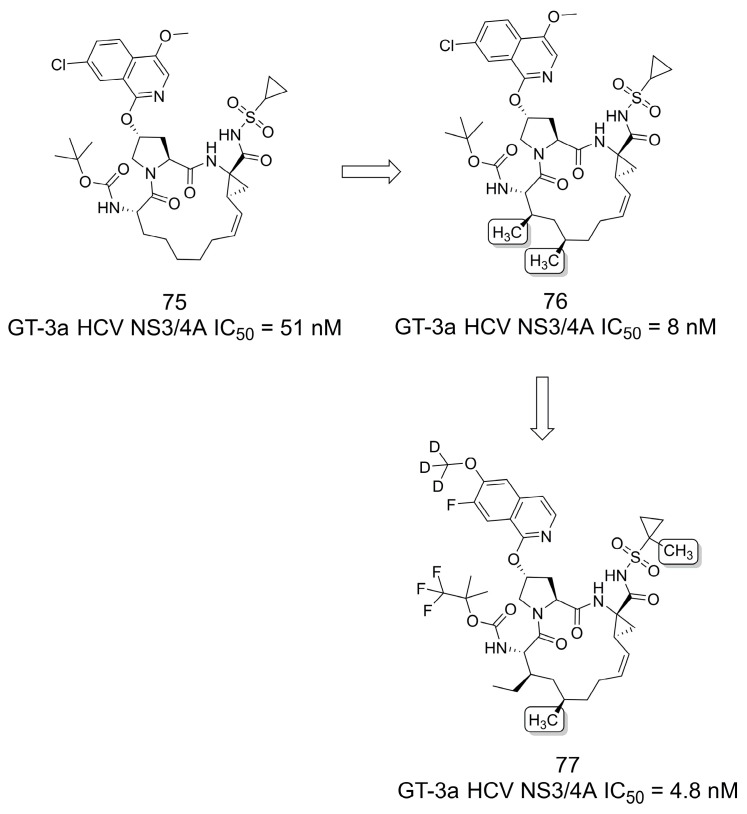
The methylation effect on NS3/4A protease inhibition for the treatment of hepatitis C virus infection [66].

The results reported by Sun and colleagues [66] highlight the effect of the methyl group in improving bioavailability following oral administration to rats of pan-genotype NS3/4A protease inhibitors for the treatment of hepatitis C virus infection [67]. First, the authors incorporated two methyl groups on **75** (IC_50_ = 51 nM) to produce **76**, a compound with improved activity (IC_50_ = 8 nM) against the genotype 3a (GT-3a) NS3/4A protease (Figure 21). Based on this compound, a series of macrocycles were designed to obtain a better in vivo profile. The authors addressed the metabolic liability of **76** by exploring the deuteration strategy and highlighted that an optimal profile was obtained by incorporating a CF_3_ into the Boc group and an additional methyl next to the polar acylsulfonamide moiety (**77**, IC_50_ = 4.8 nM). These modifications led to improvements in both in vivo distribution and metabolic stability [66].

### 3.16. Class I Histone Deacetylase (HDAC) Inhibitors

**Figure 22:**
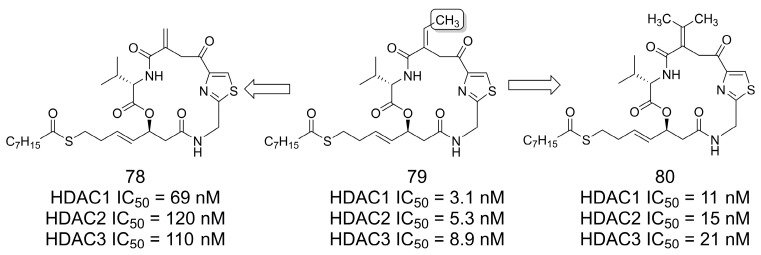
The methylation effect in the discovery of macrocyclic Class I HDAC inhibitors [68].

The authors of [68] investigated the impact of the presence of the methyl group in the design of selective Class I HDAC inhibitors as interesting candidates for cancer treatment. For example, in the macrocyclic prototype **79** (HDAC1-3 range of IC_50_ = 3.1–8.9 nM) [69], removal of the methyl group from the propenyl group resulted in compound **78** (Figure 22). Removal of the methyl group (**78**) was detrimental, resulting in IC_50_ activities in the range of 69–110 nM (HDAC1-3). When a second methyl group was added (**80**), a small decrease in the inhibitory activity was observed (HDAC1-3 range of IC_50_ = 11–21 nM). Theoretical modeling studies suggested that the binding pocket better fits the dehydrobutyrine moiety of **79**, which contains only one methyl group in the olefin subunit and seems to be important for the inhibition of HDACs from Class I [68].

### 3.17. Trypanocidal Analogs of Benznidazole

**Figure 23:**
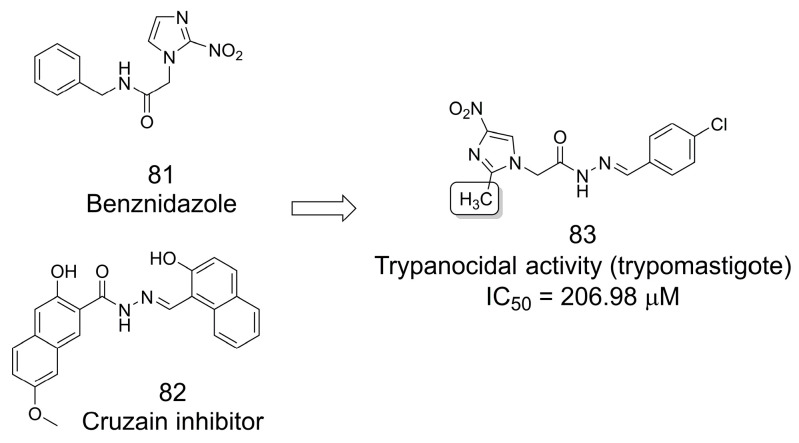
The methylation effect in the design of trypanocidal agents [70].

To design new analogs of benznidazole (**81**), Alcantara and coworkers [70] made changes to the imidazole ring, moving the nitro group to position 4 and incorporating the methyl group in position 2 (Figure 23). The authors added the methyl group based on studies showing that potency and solubility could be improved, and they moved the nitro group to position 4 based on results suggesting that such derivatives are non-toxic. In addition, the authors performed molecular hybridization based on the *N*-acylhydrazone cruzain inhibitor **82** (IC_50_ = 0.6 μM). The imidazole-*N*-arylhydrazone hybrids were tested against trypomastigote forms, and the results showed that the 4-chlorophenyl derivative (**83**) had the best trypanocidal activity with an IC_50_ of 206.98 μM [70].

### 3.18. Antibacterial Agents

**Figure 24:**
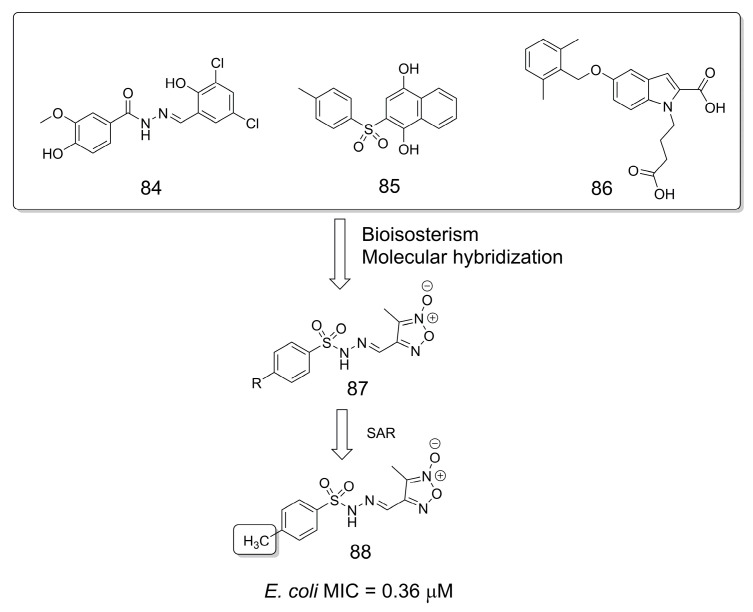
Design of sulfonylhydrazone derivatives as antibacterial agents [72].

Based on previously described β-ketoacyl acyl carrier protein synthase (FabH) inhibitors, compounds **84**–**86** [71], a series of furoxan-sulfonylhydrazone derivatives (**87**) were designed as new antibacterial agents (Figure 24) [72]. From the SAR studies, compound **88** was identified as the most potent of the series, in which the methyl group proved to be an important structural feature compared to other substituents [72].

### 3.19. Phosphonate Derivatives as Anticancer Agents

**Figure 25:**
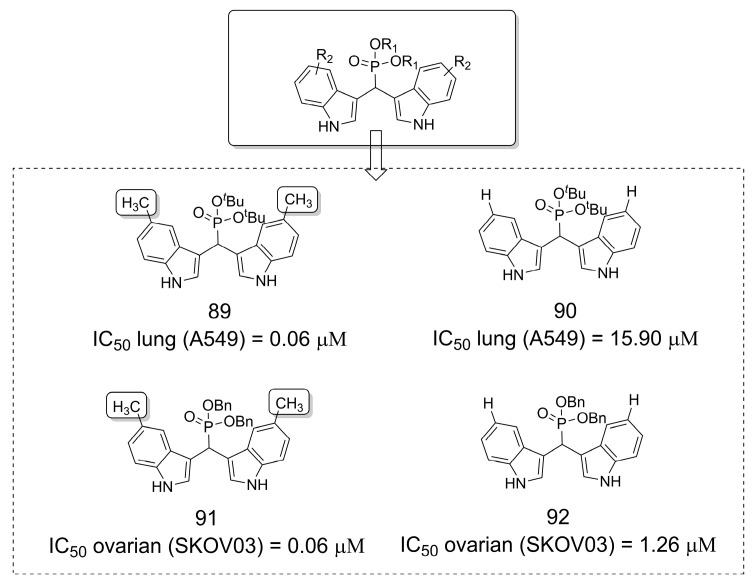
Evaluation of bis-(3-indolyl)methane phosphonate derivatives as anticancer agents [73].

A series of bis-(3-indolyl)methane phosphonate derivatives were synthesized as anticancer agents (**89**–**92**). Overall, compounds methylated at position 5 of the bis-indole core (**89** and **91**) showed increased potency for inhibiting cell proliferation of ovarian and lung cancer cell lines compared to unmethylated analogs (**90** and **92**) (Figure 25) [73].

## 4. The Methylation Effect in Physicochemical and Pharmacokinetic Property Optimization

### 4.1. Methylation Effect on Aqueous Solubility

**Figure 26:**
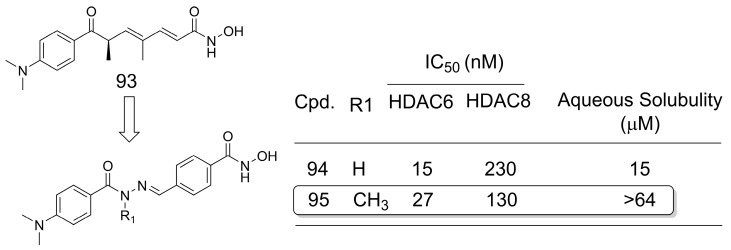
Methylation effect in *N*-acylhydrazone derivatives for aqueous solubility optimization [74].

A series of *N*-acylhydrazone derivatives were designed as HDAC6/8-selective inhibitors for cancer treatment [74]. The series was designed from the natural product trichostatin A (**93**) using bioisosteric replacement [6,7] and conformational restriction [5] strategies. The most potent compounds in the series were **94** and **95**, which differed structurally by a single methyl group (Figure 26). In this case, the magic methyl did not significantly change the activity, but the aqueous solubility was significantly increased by its presence [74], which is probably a consequence of the strong conformational effect caused by the *N*-methylation of *N*-acylhydrazone derivatives [75].

### 4.2. Methylation Effect on Plasma Stability

**Figure 27:**
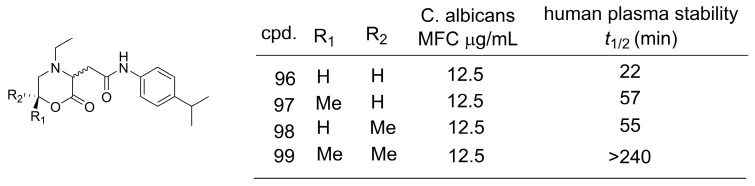
The use of the methylation effect for plasma stability optimization [76].

In the next case study, morpholin-2-one derivatives (**96**–**99**) were identified as fungicidal agents against *Candida* and *Aspergillus* species (Figure 27) [76]. However, the development of this series was hampered by low plasmatic stability, probably related to lactone hydrolysis. The introduction of methyl groups at the 6-position of the morpholin-2-one scaffold (**96**–**99**) led to a significant improvement in plasmatic stability while maintaining in vitro antifungal activity. The gemdimethyl derivative **99** was the most stable derivative as a consequence of the higher steric hindrance of lactone hydrolysis [76].

### 4.3. Methylation Effect on hERG Potassium Channel Inhibition

**Figure 28:**
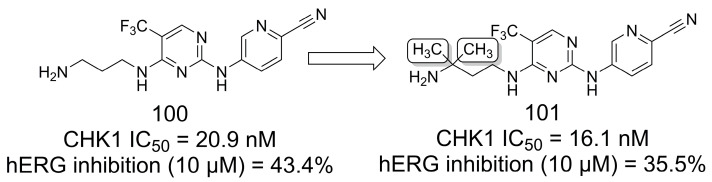
The exploration of the methylation effect for hERG inhibition profile optimization of CHK1 inhibitors [77].

Jin and coworkers [77] reported the introduction of methyl groups into the aminopropylamine chain of compound **100** (checkpoint kinase 1—CHK1 IC_50_ = 20.9 nM) to provide a series of CHK1 inhibitors. These compounds showed excellent inhibitory activity, and compound **101** was the most potent (CHK1 IC_50_ = 16.1 nM). Additionally, **101** showed reduced inhibition of the human ether-à-go-go-related (hERG) potassium channel (35.5% at 10 μM) compared to **100** (43.4% at 10 μM) (Figure 28). Furthermore, the authors suggested that the introduction of the gem-dimethyl group improved *in vivo* metabolic stability compared to linear amines [77].

**Figure 29:**
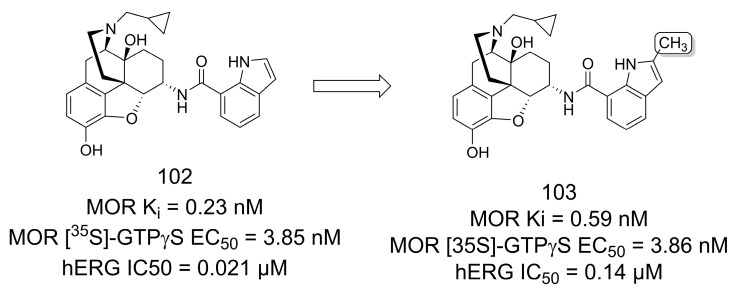
The methylation effect in hERG inhibition profile optimization of mu opioid ligands [78].

In another study, Ma and colleagues [78] designed analogs of the mu opioid receptor (MOR) ligand NAN (**102**) [79], a 6α-*N*-7′-indolyl-substituted naltrexamine derivative, which showed promising pharmacological effects but had significant hERG potassium channel liability (Figure 29). According to *in vivo* morphine-induced antinociception assays, compound **103** was the most potent antagonist. This compound (**103**) bears a methyl group at the 2′ position of the indole ring and had a sevenfold lower potency for hERG potassium channel inhibition compared to NAN (**101**) [78].

### 4.4. Methylation Effect on Metabolism

**Figure 30:**
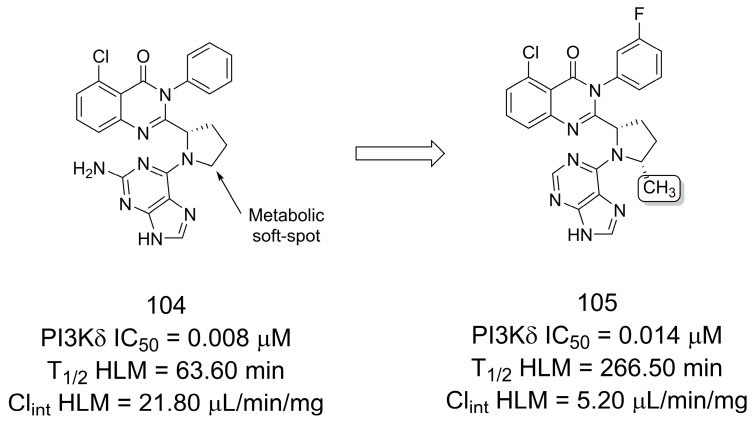
Metabolic profile optimization using the methylation effect [80].

Liu and coworkers [80] reported the modulation of linkers of phosphoinositide 3-kinase delta inhibitors and found that by introducing the “magic methyl” group they had the best balance between oxidative metabolism, stability, and potency. The quinazolinone derivative **104** showed significant inhibitory potency on PI3Kδ with an IC_50_ value of 0.008 μM (Figure 30). However, compound **104** showed a high clearance with a Cl_int_ value of 21.80 µL/mg/min in human liver microsomes (HLMs). Additional metabolite identification studies of compound **104** revealed that oxidation of the five-membered pyrrolidine linker was the main soft spot for metabolic reactions. This led to the design of new analogs of **104**, resulting in compound **105**, which demonstrated favorable bioavailability in Sprague-Dawley rats following intravenous and oral treatment. In addition, compound **105** had a PI3Kδ IC_50_ of 0.014 μM and activated basophils and B cells and was effective in a collagen-induced arthritis model [80].

## 5. Perspectives

Analyzing the state of the art in the use of the methyl effect in medicinal chemistry, it is evident that its applicability to the discovery and optimization of new small-molecule drug candidates is indisputable. In this review, the importance of this group for improving pharmacodynamic properties has been discussed, highlighted by the discovery of the recently approved anticancer drug tazemetostat (**8**), where the authors found that four methyl groups inserted at different positions resulted in a stunning >100,000-fold improvement in activity. Indeed, there are many examples focusing on the effect of methylation on the pharmacodynamic properties of bioactive molecules. However, in this review some examples of the influence of the methyl group on the pharmacokinetic and physicochemical profile of drug candidates have been presented, covering its use to block metabolic soft spots, reduce hERG liability, improve aqueous solubility, and increase plasma stability. From its participation in the molecular recognition process of pharmacological targets to the modulation of ADMET properties, the “magic methyl” never ceases to surprise us. We hope that the key examples discussed here will help the scientific community to further understand either the relationship between the structure and biological activity of new chemical entities or the rational application of methylation and what can be expected from this process.
